# Surgical Wait Time Is Not Associated With Oncological or Psychosocial Outcomes After Robotic Radical Prostatectomy

**DOI:** 10.1155/proc/4314397

**Published:** 2025-07-29

**Authors:** Juliette Cotte, Scott Leslie, Jacob Bird, Patrick-Julien Treacy, Nicholas Hirst, Kate Alexander, Daniel Steffens, Ruban Thanigasalam

**Affiliations:** ^1^Department of Urology, Royal Prince Alfred Hospital, Camperdown, New South Wales, Australia; ^2^Department of Urology, Chris O'Brien Lifehouse, Camperdown, New South Wales, Australia; ^3^Surgical Outcomes Research Centre (SOuRCe), Royal Prince Alfred Hospital, Camperdown, New South Wales, Australia; ^4^Institute of Academic Surgery, Royal Prince Alfred Hospital, Camperdown, New South Wales, Australia; ^5^Department of Urology, Concord Repatriation General Hospital, Concord, New South Wales, Australia; ^6^University of Sydney, Camperdown, New South Wales, Australia

**Keywords:** oncological outcomes, prostate cancer, quality of life, radical prostatectomy, surgical wait time

## Abstract

**Background:** Prostate cancer (PCa) is a prevalent malignancy in men, with increasing incidence and longer wait times for curative surgery, particularly in public health systems. While the impact of surgical wait time (SWT) on oncological outcomes in PCa remains controversial, its influence on patient-reported outcomes has not been thoroughly evaluated.

**Objective:** To assess the impact of SWT on both oncological and psychological outcomes in patients undergoing robot-assisted radical prostatectomy (RARP) for preoperative ISUP grade 2 and 3 PCa.

**Methods:** This retrospective single-center study included patients who underwent RARP for intermediate risk localized PCa between April 2016 and August 2024. Patients were stratified into two groups based on SWT: < 6 months vs. ≥ 6 months. The primary outcome was recurrence-free survival (RFS) for all patients. Secondary outcomes included RFS in a high-risk subgroup defined by pathological features (pT3 stage, seminal vesicle invasion, extracapsular extension, and positive surgical margins), as well as a comparison of functional outcomes between the two groups. Patient-reported outcomes were evaluated using SF-36 (mental and physical components) and the Decision Regret Scale (DRS) at 6 weeks, 3 months, 6 months, and 1 year. Statistical analyses included Kaplan–Meier survival estimates, Cox proportional hazard models, and comparative tests with *p* < 0.05 considered significant.

**Results:** 218 patients have been included. RFS did not significantly differ between groups (*p*=0.98), including among high-risk patients (*p*=1.00). No significant differences were found in extraprostatic extension, seminal vesicle invasion, positive surgical margins, or ISUP upgrading between groups. Similarly, changes in both SF-36 physical and mental and DRS scores showed no statistically significant differences at all time points.

**Conclusion:** In this cohort of patients with intermediate-risk PCa, SWT beyond 6 months did not adversely affect oncological or health-related quality of life outcomes.

## 1. Introduction

Prostate cancer (PCa) is one of the most common malignancies in men worldwide. In Australia, nearly 23,500 new cases are diagnosed each year, with incidence rising over the past decade [1]. Additionally, the number of PCa patients with organ-confined disease is also increasing [2]. This trend has led to longer wait times for surgery, particularly for robot-assisted procedures in the public system. The impact of surgical wait time (SWT)—defined as the interval between diagnosis and curative treatment—on oncological and survival outcomes has been reported in several cancers, including bladder [3], colorectal [4, 5], and breast cancer [6]. However, studies on PCa have yielded conflicting results. Some have found that SWT between prostate biopsy and radical prostatectomy does not adversely affect biochemical recurrence (BCR) or pathological outcomes [7–9], even in high-risk patients [10]. Conversely, other studies suggest that prolonged SWT negatively impacts BCR and pathological outcomes [11, 12]. Luu et al. found that delaying surgery beyond 6 months after diagnosis did not significantly affect adverse pathological features or overall survival [13]. In contrast, O'Brien et al. reported that, in men meeting the D'Amico low-risk criteria, a surgical delay of 6 months or more was associated with significantly worse radical prostatectomy outcomes [11]. Finally, Nguyen et al. suggested in a meta-analysis that there is a higher risk of BCR and worse pathological outcomes associated with delays beyond 6–9 months [14]. Thus, the impact of SWT remains controversial. Moreover, none of these studies have examined the effect of SWT on patient-reported outcomes (PROMs), despite evidence that a cancer diagnosis affects patients' quality of life [15–17]. Vyas et al. reported that radical treatments for PCa significantly impact mental and social well-being [18]. However, there is a lack of literature on the psychosocial impact of delays between diagnosis and surgical management in PCa. The aim of this study is to determine the impact of SWT on both oncological and psychological outcomes following robot-assisted radical prostatectomy (RARP).

## 2. Materials and Methods

### 2.1. Study Design

This is a retrospective, single-center study including consecutive patients who underwent RARP in a public teaching hospital in Sydney, Australia, between April 2016 and August 2024. Patients with ISUP grade 2 and 3 on initial prostate biopsy, as well as those with a follow-up period of more than one year, were included. Patients with ISUP 1, 4, and 5 were excluded. SWT was defined as the period between the date of prostate biopsy and the date of surgery. Patients were divided into two groups based on SWT: less than 6 months and more than 6 months.

### 2.2. Description of the Procedure

The Da Vinci Xi from Intuitive Surgical was used to carry out the RARP. Four expert consultant surgeons conducted all the procedures, while trainee surgeons were supervised in performing certain parts of the surgery. A transperitoneal anterior multiport approach was performed for all the patients. Relevant specimens collected during surgery were sent to the pathology department for histopathological analysis. Histopathological analysis for each specimen was then performed by an expert uropathologist. Gleason score was reported based on standardized ISUP grade groups. The presence of seminal vesicle invasion (SVI) and extracapsular extension (ECE) was evaluated separately and detailed in each histopathology report. Additionally, the reports included information on surgical margins and overall disease staging.

### 2.3. Data Collection

The following data were collected from each patient:

- Clinical and demographic characteristics: sex, age, and BMI at the time of surgery for RARP; preoperative PSA; date of prostate biopsy; ISUP grade group on prostate biopsy; and presence of MRI performed and PIRADS score, if available.
- Intervention characteristics: date of surgery and pathology report (ISUP grade group, upgrade in ISUP grade compared to previous prostate biopsy report, SVI, extracapsular prostatic extension (EPE), presence of positive margins, and T score on pathology report).
- Outcomes: recurrence-free survival (RFS) based on PSA (measured at 6 weeks, 3 months, 6 months, 1 year, 2 years, 3 years, and 5 years) and PROMs assessed by the physical and mental SF-36 questionnaire (sent by mail at 6 weeks, 3 months, 6 months, and 1 year) and by the Decision Regret Scale (DRS) (sent by mail at 6 weeks, 3 months, and 1 year).

### 2.4. Outcomes

Our primary outcome was the comparison of RFS, with recurrence defined as a PSA > 0.2 at any time during the follow-up, between patients who underwent surgery within 6 months or more than 6 months after prostate biopsy.

The secondary outcomes were as follows:

- Comparison of RFS, in the subgroup of high risk patients on the prostatectomy specimen, defined as EPE+, SVI+, pT3 on prostatectomy reports, and an upgrade in ISUP grade group.
- Comparison of health-related quality of life (HRQOL) outcomes, defined by the results of the physical and mental SF-36 and DRS questionnaires.

The last follow-up was determined by the most recent date we were able to receive news on the patient.

### 2.5. Statistical Analysis

Our objective was to determine the impact of SWT on both oncological and psychological outcomes following RARP. General and intervention characteristics were described by frequencies and percentages for categorical variables and by mean±standard deviation (SD) or median (Q1–Q3) for continuous variables. The Kaplan–Meier method was used to describe RFS, from the date of surgery to recurrence, defined by PSA > 0.2 ng/mL at any time during the follow-up or end of follow-up (date of last visit). Patients without PSA recurrence were censored at the date of the last follow-up. Differences between the 2 groups of SWT were analyzed using a Cox model. The chi-square test was used to compare categorical variables, and the T-test was used to compare continuous variables. *p* values < 0.05 were considered to be statistically significant. All analyses were performed using R software (Version 3.6.2) [19].

### 2.6. Ethics

Ethics approval was granted by the Sydney Local Health District Human Ethics Committee (X16-0294 & 2019/ETH06594). This study followed the Strengthening of Reporting Observational Studies in Epidemiology (STROBE) guidelines [20].

## 3. Results

### 3.1. Study Population

**Figure 1:**
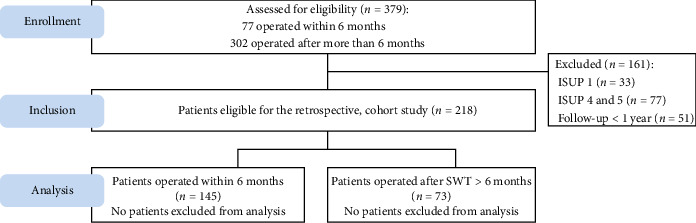
Flow diagram.

A total of 379 consecutive patients underwent RARP between April 2016 and August 2024. 161 patients were excluded: 33 due to ISUP grade 1 and 77 due to ISUP grades 4 and 5. Among this 269, 51 were excluded due to a follow-up period of less than one year. Consequently, 218 patients were eligible for this study. Among them, 145 patients had a SWT of less than six months between biopsy and surgery, while 73 patients waited more than six months (Figure 1).

### 3.2. Description of Patients' Characteristics

Mean age at surgery was 64.3 years (SD 8.1) for men operated on within 6 months and 66.3 years for those operated on after 6 months (SD 7.0). Mean preoperative PSA was 8.5 (SD 5.5), with no significant difference between the two groups. Mean follow-up duration was 37.2 months (SD 20.0): 39.04 months (SD 19.1) for men operated on within 6 months and 33.6 months (SD 21.6) for those operated on after 6 months. A preoperative MRI was performed in 81.2% of patients, with no significant difference in PIRADS scores between the two groups. Prostate biopsy reported Gleason 7 (3 + 4) in 66.2% and Gleason 7 (4 + 3) in 33.8% of men operated on within 6 months, compared, respectively, to 75.3% and 24.6% of those operated on after 6 months. However, this difference was not statistically significant (Table 1).

### 3.3. Outcomes

**Figure 2:**
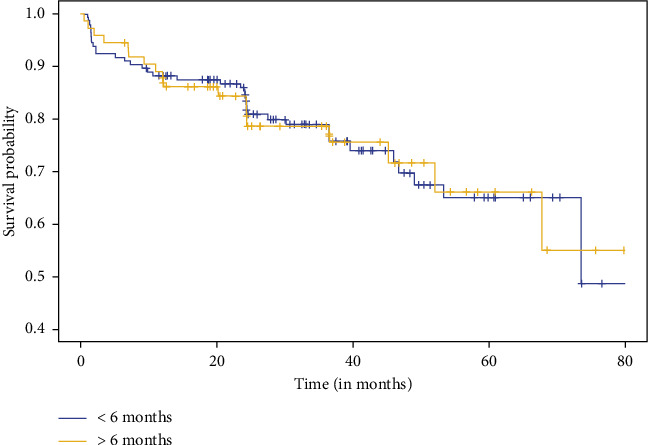
Recurrence-free survival depending on SWT.

**Figure 3:**
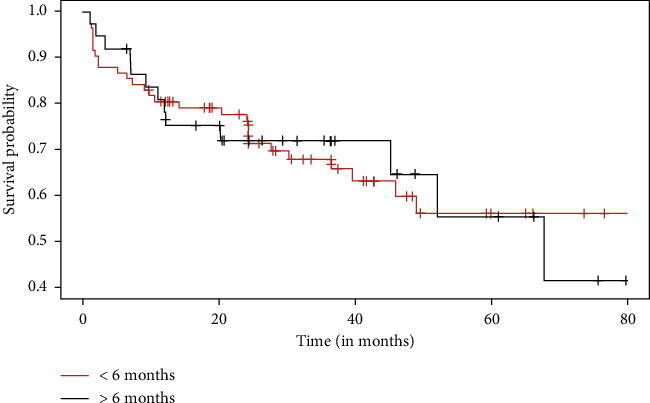
Recurrence-free survival for high-risk patients (pT3, upgrade Gleason, SVI+, and EPE+) depending on SWT.

**Figure 4:**
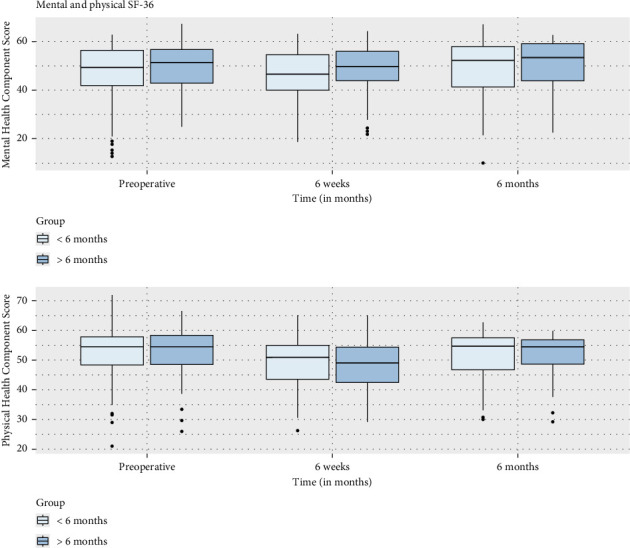
Comparison of mental and physical SF-36 scores preoperatively at 6 weeks and at 6 months postoperative between patients operated within 6 months versus after 6 months of SWT.

**Figure 5:**
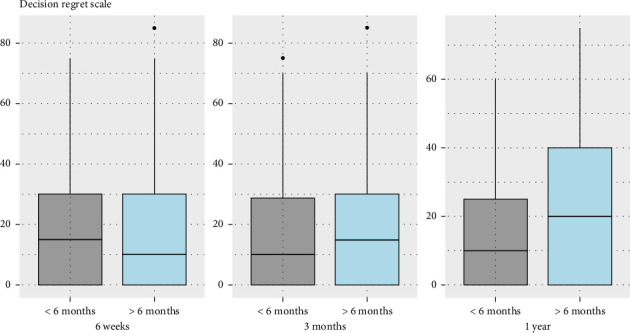
Comparison of evolution in Decision Regret Scale results between patients operated within 6 months of after 6 months of SWT.

There was no significant difference in RFS between the two groups (*p* = 0.98) (Figure 2), even when we compared the difference in survival between patients with high risk of recurrence (*p* = 1.00) (Figure 3). Regarding pathology reports, the rates of EPE and SVI were similar between the two groups, with no significant differences (*p* = 0.89 and *p* = 0.47, respectively). Positive surgical margins were observed in 29.4% of men operated on within 6 months (11.9% positive margins in pT2, 50% in pT3) and 27.6% of those operated on after 6 months (*p* = 0.42). The distribution of *T*-scores showed no significant variation between groups, with most patients classified as T2a or T3a. Gleason upgrading occurred in 21.1% of cases, with no significant difference between groups (*p* = 0.62). Among 218 patients, 57% with a SWT of less than six months had recurrence risk factors (pT3, EPE, SVI, and upgrade Gleason on specimen pathology), compared to 51% in the group with SWT longer than six months, with no significant difference between the two groups (*p* = 0.41) (Table 2). Regarding impact on quality of life, the analyses comparing changes in Mental and Physical Health Component Scores (MHCS and PHCS) at 6 weeks and 6 months between the two groups showed no statistically significant differences. At 6 weeks, both MHCS (*p* = 0.4377) and PHCS (*p* = 0.5309) changes were similar between groups. Similarly, at 6 months, neither MHCS (*p* = 0.3952) nor PHCS (*p* = 0.7015) demonstrated significant differences (Figure 4). The comparison of DRS scores between the two groups at different time points showed no significant differences. At 6 weeks, the mean scores were nearly identical (*p* = 0.994). At 1 year post-RARP, DRS scores in patients waiting more than 6 months compared to those waiting less than 6 months also showed no significant difference (*p* = 0.06) (Figure 5).

### 3.4. Supporting Information Description

Two post hoc analyses were performed. First, a sensitivity analysis using 3-, 6-, and 9-month thresholds showed no significant differences (*p*=0.39) (see Supporting Information 1—Additional analyses using 3-, 6-, and 9-month surgical wait time thresholds). Second, SWT was analyzed as a continuous variable in a Cox proportional hazards model, which also showed no significant association with RFS (*p*=0.42) (see Supporting Information 2—Analysis using SWT as a continuous variable in a Cox proportional hazard model).

## 4. Discussion

Our study aimed to evaluate the impact of SWT on oncological and psychological outcomes in patients undergoing RARP for PCa. In line with the findings of the ProtecT trial [21], which reported similar long-term survival outcomes across active monitoring, surgery, and radiotherapy, our results support the idea that timely, but not urgent, intervention may be appropriate for many patients with localized disease.

From an oncological perspective, our results align with previous studies suggesting that a prolonged SWT does not significantly alter BCR rates or pathological features such as EPE, SVI, or positive surgical margins. This supports the notion described by Hisawara et al. that, in carefully selected patients, a moderate delay in surgery may not compromise cancer control. It is also consistent with Nguyen et al.'s meta-analysis that also found that treatment delays up to 3 months are safe for all localized PCas, and even delays beyond 6–9 months were inconsistently associated with adverse outcomes—limited to increased risk of BCR and worse pathology, but not impacting cancer-specific or overall survival [14]. However, these findings contrast with studies reporting a negative impact of delayed treatment on oncological outcomes such as studies published by Abern et al. in 2013 [12] or O'Brien et al. in 2011 [11], emphasizing the need for further research with larger cohorts to clarify potential subgroup effects.

In our cohort, we excluded patients classified as ISUP 1, 4, and 5. Specifically, ISUP 1 patients were excluded due to the fact that many were initially under active surveillance. Additionally, a significant portion of these patients did not undergo a new biopsy prior to surgery, with progression only being observed on MRI. The waiting times for surgery were notably long but were not related to issues with access to surgical intervention. On the other hand, patients classified as ISUP 4 and 5 were predominantly operated on within 3 months, with significantly lower RFS rates. Given that the primary objective of this study was to evaluate access to operating rooms, we chose to retain a more homogeneous group.

A 6-month SWT cutoff has been chosen for several reasons. Firstly, it is the most commonly reported threshold in the literature, whether showing a difference (O'Brien et al., J Urol, 2011) or not (Luu et al., Clin Genitourin Cancer, 2024) [8, 11–13]. Moreover, this timeframe reflects clinical practice, where most urologists in our department consider delays of up to 6 months as having no meaningful impact on surgical outcomes. From a practical standpoint, defining SWT in terms of discrete intervals provides clearer guidance for clinicians when scheduling surgeries and communicating with patients.

Regarding psychosocial outcomes, our study is among the few to assess PROMs in the context of surgical delays. Changes in both the MHCS and PHCS from the SF-36 questionnaire did not differ significantly between the two groups at either 6 weeks or 6 months postoperatively. This suggests that, at least in the short to medium term, delaying surgery does not have a substantial effect on patients' perceived quality of life. However, the observed trend of higher DRS in patients with longer SWT suggests that prolonged waiting may contribute to increased dissatisfaction with treatment timing, even if clinical outcomes remain comparable. While these differences did not reach statistical significance, they highlight the importance of addressing patient expectations and psychological distress associated with waiting for surgery.

Our study has several strengths, including a well-defined cohort, standardized surgical techniques, and comprehensive oncological and psychosocial follow-up. However, limitations include the retrospective nature of the study, the short follow-up (mean around 3 years), and the relatively small sample size, which may limit the detection of subtle differences between groups. As prospective studies seem unfeasible—since SWT is not an assignable exposure—future research should ideally focus on larger cohort studies to better elucidate the potential impact of SWT on patient outcomes.

## 5. Conclusion

In conclusion, our findings suggest that a SWT beyond 6 months does not adversely affect oncological or quality-of-life outcomes in selected men undergoing RARP for PCa. Further studies should be conducted, particularly to assess the impact on quality of life, which remains underrepresented in the literature.

## Figures and Tables

**Table 1:** Patients' characteristics.

Demographics	Overall cohort	Waiting time < 6 months	Waiting time ≥ 6 months	Missing data *N* (%)	*p* value
No. of patients	218	145	73	—	
Age, years (mean, SD)	64.97 ± 7.73	64.32 ± 8.10	66.27 ± 6.79	—	0.06
Mean follow-up (in months) (mean, SD)	37.22 ± 20.03	39.04 ± 19.14	33.6 ± 21.38		0.07
BMI (mean, SD)	27.45 ± 4.42	27.58 ± 4.4	27.19 ± 4.49	3 (0.013%)	0.55
Pre-op PSA (mean, SD)	8.48 ± 5.53	8.55 ± 5.95	8.33 ± 4.61	2 (< 0.01%)	0.78
MRI performed (*n*, %)	177 (81.2%)	115 (79.3%)	62 (84.9%)	—	0.31
PIRADS (*n*, %)				3 (1.7%)	
1	5 (2.8%)	3 (2.6%)	2 (3.2%)		0.06^∗^
2	18 (10.2%)	8 (7.0%)	10 (16.1%)		
3	28 (15.8%)	14 (12.2%)	14 (22.6%)		
4	70 (39.5%)	48 (41.7%)	22 (35.5%)		
5	53 (29.9%)	39 (33.9%)	14 (22.6%)		
Gleason score on prostate biopsy (*n*, %)					
3 + 4	151 (69.3%)	96 (66.2%)	55 (75.3%)	—	0.17
4 + 3	67 (30.7%)	49 (33.8%)	18 (24.6%)	—	

^∗^Fisher test.

**Table 2:** Pathology outcomes.

Pathology (clinical) outcomes		< 6 months	≥ 6 months	Missing data *N* (%)	*p* value
EPE	94 (43.1%)	63 (43.5%)	31 (42.5%)	—	0.89
SVI	19 (8.7%)	12 (8.3%)	7 (9.6%)	50	0.47
Positive margins	64 (29.4%)	40 (27.6%)	24 (32.9%)	—	0.42
*T*-score				—	
T2a	79 (36.2%)	54 (37.3%)	25 (34.3%)		0.12
T2b	15 (6.9%)	6 (4.1%)	9 (12.3%)		
T2c	24 (11.0%)	16 (11.0%)	8 (10.9%)		
T3a	77 (35.3%)	56 (38.6%)	21 (28.8%)		
T3b	23 (10.6%)	13 (9.0%)	10 (13.7%)		
Gleason upgrade	46 (21.1%)	32 (22.1%)	14 (19.2%)	—	0.62

## Data Availability

The data that support the findings of this study are available on request from the corresponding author. The data are not publicly available due to privacy or ethical restrictions.

## Supporting Information

Supporting Information 1 Supporting Information no. 1—Additional analyses using 3-, 6-, and 9-month surgical wait time thresholds.

Supporting Information 2 Supporting Information no. 2—Analysis using SWT as a continuous variable in a Cox proportional hazard model.
