# Supplementary figures and images for: Surgical Wait Time Is Not Associated With Oncological or Psychosocial Outcomes After Robotic Radical Prostatectomy
Source: Prostate Cancer. 2025 Jul 29;2025:4314397. doi: 10.1155/proc/4314397 (PMC12324907; doi:10.1155/proc/4314397)

## Recurrence after RALP depending on SWT

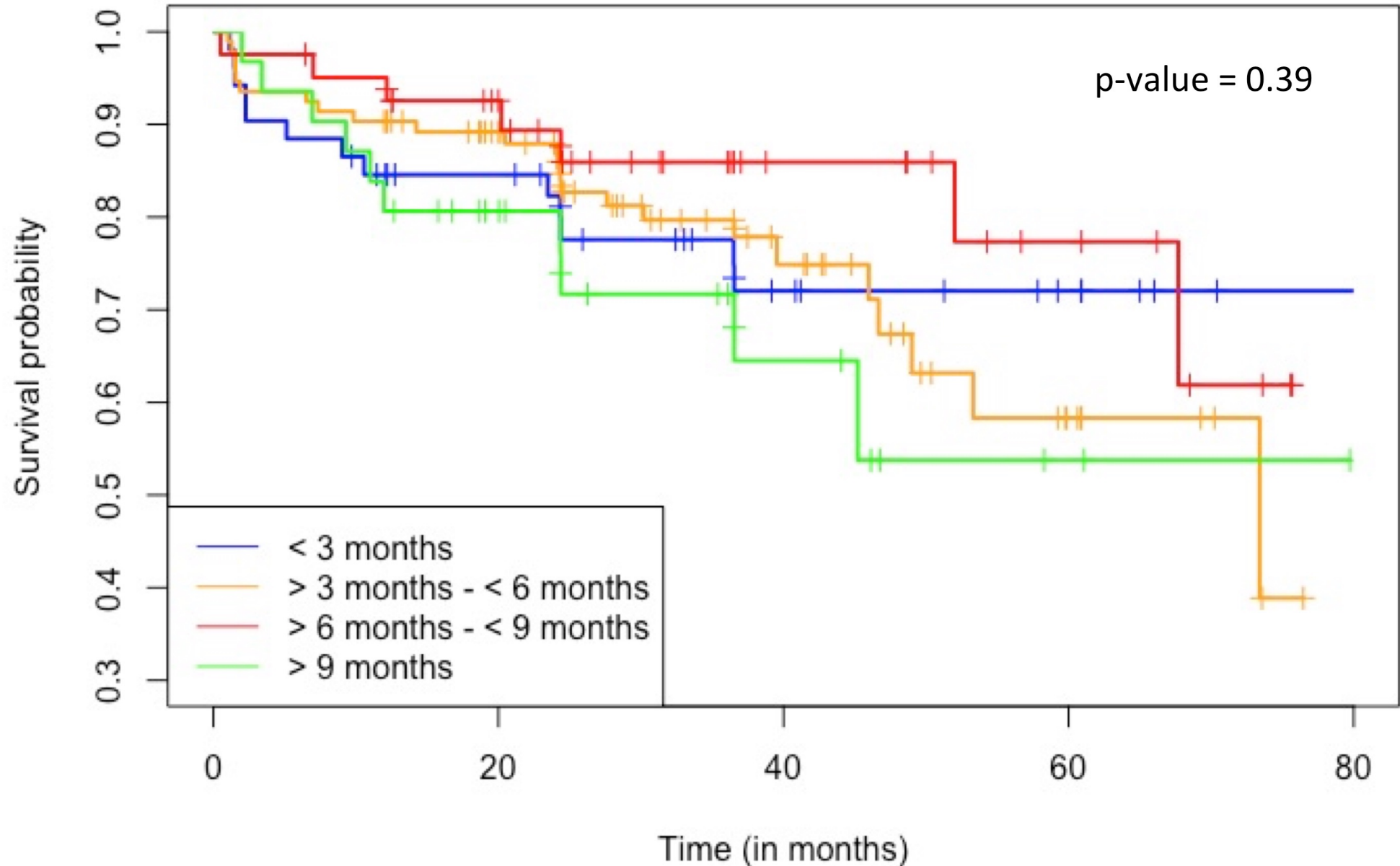

Supplement: Supporting Information 1 — Supporting Information no. 1—Additional analyses using 3-, 6-, and 9-month surgical wait time thresholds. [file 4314397.f1.pdf]

### Recurrence-free survival

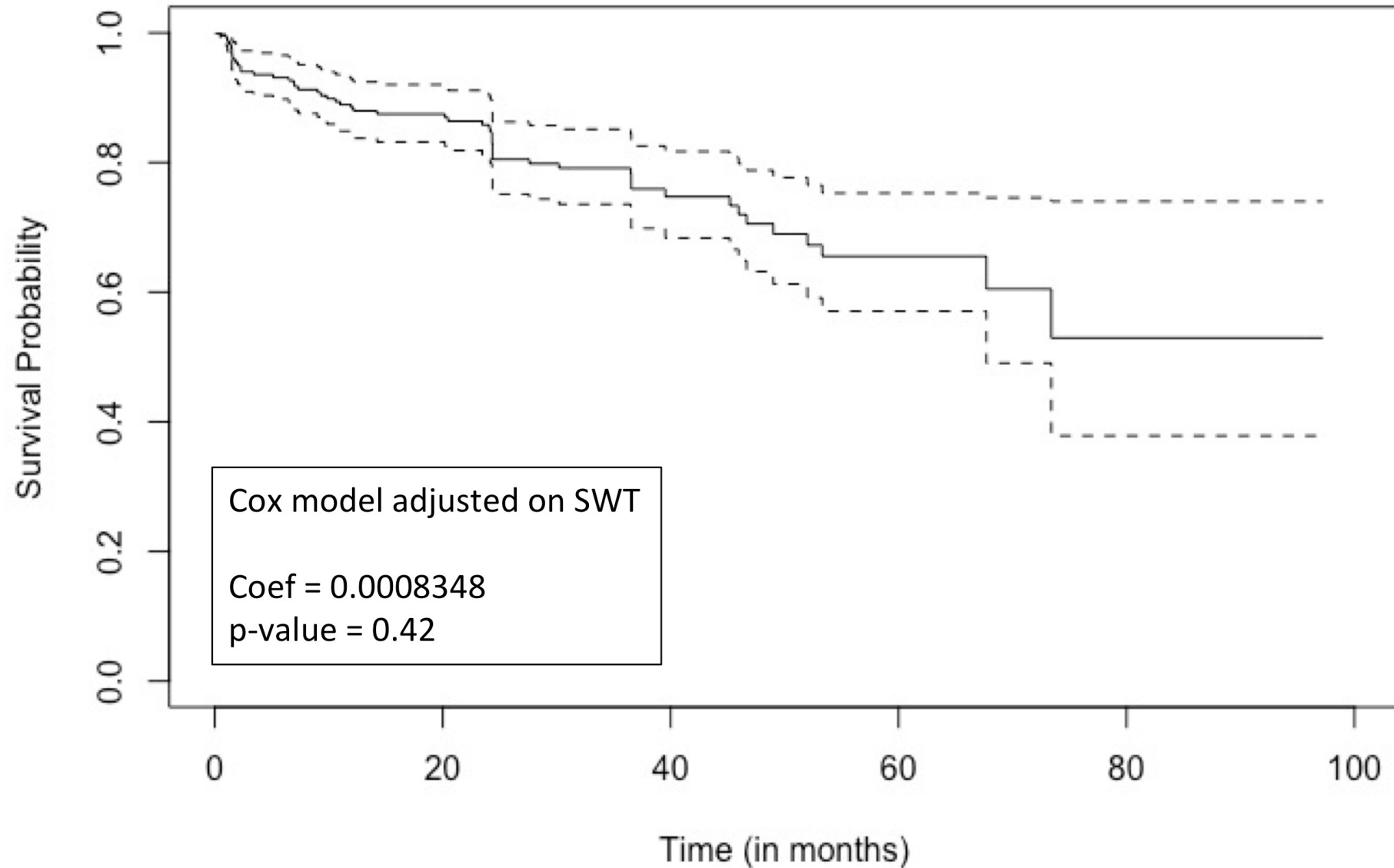

Supplement: Supporting Information 2 — Supporting Information no. 2—Analysis using SWT as a continuous variable in a Cox proportional hazard model. [file 4314397.f2.pdf]
